# The Inflammatory Footprint of Anti-Breast Cancer Treatments and Psychosocial Factors in Women Undergoing Chemotherapy

**DOI:** 10.3390/biomedicines13102563

**Published:** 2025-10-21

**Authors:** Magda A. Oliveira, Susana S. Almeida, Gabriela Martins, Inês Godinho, Carlos Palmeira, Maria Emília Sousa, Lia Fernandes, Rui Medeiros, Marina Prista Guerra

**Affiliations:** 1 Department of Medicine, Psychology Service, Portuguese Oncology Institute of Porto (IPO Porto), Medical Oncology Group, Research Center of IPO Porto (CI-IPOP)/RISE@CI-IPOP (Health Research Network), Rua Dr. António Bernardino de Almeida 865, 4200-072 Porto, Portugal; 2 Faculty of Psychology and Educational Sciences (FPCEUP), University of Porto, Rua Alfredo Allen, 4200-135 Porto, Portugal; mguerra@fpce.up.pt; 3 CUF Porto Hospital, Estrada da Circunvalação 14341, 4100-180 Porto, Portugal; susanasalmeida@ipoporto.min-saude.pt; 4 Department of Medicine, Psychiatry Service, Portuguese Oncology Institute of Porto (IPO Porto), Research Center of IPO Porto (CI-IPOP)/RISE@CI-IPOP (Health Research Network), Rua Dr. António Bernardino de Almeida, 865, 4200-072 Porto, Portugal; 5 RISE-Health, Department of Clinical Neurosciences and Mental Health, Faculty of Medicine, University of Porto, Alameda Prof. Hernâni Monteiro, 4200-319 Porto, Portugal; 6 Clinical Pathology Service, Portuguese Oncology Institute of Porto (IPO Porto), Rua Dr. António Bernardino de Almeida 865, 4200-072 Porto, Portugal; gmartins@ipoporto.min-saude.pt (G.M.); mimmmg@gmail.com (I.G.); carlospalmeira@ipoporto.min-saude.pt (C.P.); emilia.sousa@ipoporto.min-saude.pt (M.E.S.); 7 RISE-Health, Department of Clinical Neurosciences and Mental Health, Faculty of Medicine, University of Porto, Psychiatry Service, São João University Hospital, Porto, Alameda Prof. Hernâni Monteiro, 4200-319 Porto, Portugal; lfernandes@med.up.pt; 8 Molecular Oncology and Viral Pathology Group, Research Center of IPO Porto (CI-IPOP)/RISE@CI-IPOP (Health Research Network), Portuguese Oncology Institute of Porto (IPO Porto)/Porto Comprehensive Cancer Center (Porto.CCC), Research Center-LAB2, Rua Dr. António Bernardino de Almeida 865, 4200-072 Porto, Portugal; ruimedei@ipoporto.min-saude.pt; 9 ICBAS, Abel Salazar Institute for the Biomedical Sciences, University of Porto, Rua Jorge Viterbo Ferreira 228, 4050-313 Porto, Portugal; 10 Biomedical Research Center (CEBIMED), Faculty of Health Sciences, Fernando Pessoa University (UFP), Praça 9 de Abril 349, 4249-004 Porto, Portugal; 11 Research Department, LPCC-Portuguese League Against Cancer (NRNorte), Estrada da Circunvalação 6657, 4200-172 Porto, Portugal; 12 Center for Psychology at University of Porto (CPUP), Rua Alfredo Allen, 4200-135 Porto, Portugal

**Keywords:** breast cancer, cancer therapies, biomarkers, coping strategies, inflammation, psychoneuroimmunology and cancer, quality of life, stress, well-being

## Abstract

**Background/Objectives**: Despite the well-recognized role of inflammation in breast cancer course, the biological mechanisms involved in its pathophysiology are complex, heterogeneous, and still unclear. However, evidence shows that cancer treatments and stress system responses impact the patient’s inflammatory status. We aim to analyze the inflammatory footprint of anti-breast cancer treatments and psychosocial factors by observing the evolution of inflammatory and psychosocial markers pre- and post-chemotherapy; to examine the associations between pro- and anti-inflammatory cytokines with psychosocial factors after chemotherapy; and to identify vulnerability/resilience variables that may improve patients’ referral for psycho-oncological interventions before/after chemotherapy. **Methods**: We performed a well-controlled cohort study of premenopausal women diagnosed with stage I to III breast cancer undergoing chemotherapy. Patients were longitudinally evaluated at pre-chemotherapy (post-surgery in the adjuvant cohort) and post-chemotherapy. Both evaluations included clinical, immunological, and psychosocial data. **Results**: A significant decrease in TNF-α (*p* = 0.001) was observed in the adjuvant cohort compared to the neoadjuvant cohort. After chemotherapy, we found a significant decline in IL-17a, TNF-α, and IL-10 (*p* = 0.000, 0.000, 0.020), reinforcing the influence of chemotherapy on immunocompetence. Significant relations (*p* < 0.01) were found between the inflammatory biomarkers that decreased post-chemotherapy and psychosocial factors. Venting and instrumental/emotional support coping played the greatest role in immunological–psychological interactions. **Conclusions**: The findings confirm an inflammatory footprint, linking the complex interplay between breast tumors, anti-breast cancer treatments, and psychosocial factors. By supporting the immunoregulatory role of biological and psychosocial factors in immunocompetence, our findings bring potential insights into a biopsychosocial approach that targets both survival and psychological adjustment outcomes.

## 1. Introduction

Breast cancer (BC) is the most common cancer and the leading cause of cancer-related deaths in women. Therefore, reducing recurrence and improving survival while maintaining quality of life are crucial. Among the several mechanisms involved in BC etiology and progression, inflammatory factors stand out [1,2,3].

The cancer-specific inflammatory equation comprises complex, multifactorial elements, of which the following warrant particular attention.

First is tumor microenvironment (TME) characteristics. The literature suggests a dynamic relationship in which the inflammatory response depends on the TME to regulate tumor cell survival, proliferation, differentiation, activation, migration, and apoptosis [1,2,3,4,5,6], while chronic inflammation influences tumorigenesis, shaping the TME and promoting cancer risk, progression, and recurrence [1,2]. The TME strongly impacts tumor progression and clinical outcomes [3,6,7]. Tumor cells induce inflammation by activating white blood cells and releasing inflammatory cytokines (e.g., TNF-α, IL-1β, IL-6, IL-21, and TGF-β), which are critical at the tumor site [3,4,5,8]. Although cytokines are essential for synaptic plasticity and immunomodulation and for adaptive and innate immune regulation [2,4], their over-expression is associated with functional impairments. Cytokines are key regulators for BC development, influencing tumor cell behavior and reprograming tumor niche via specific signaling pathways [9]. The literature reports that Th17 cells play a key role in BC via inflammatory processes. Studies have shown that Th17 cell-secreted cytokines (IL-17, TNF-α, and IL-6) are closely linked to cancer stem cells and the TME, with significantly higher levels in the peripheral blood of BC patients than in healthy individuals. Thus, they critically suppress the immune system, disrupting inflammatory balance and promoting tumor growth, proliferation, and invasion and accelerated BC progression [3,4,8].

Second is anticancer treatments’ influence on immune response and inflammation. The literature has increasingly systematized not only the prognostic value of cytokines and their potential role as therapeutic targets (e.g., [10,11,12,13,14,15,16]), but also the direct impact of anticancer therapies on circulating cytokine concentrations. Regarding the last topic, most observational studies and trials, generally with a small sample size, report acute increases in cytokine levels following surgery and chemotherapy. However, the findings are more robust and consistently reproducible for the acute inflammatory response to surgery, whereas longitudinal and high-quality data on the direct impact of chemotherapy on cytokines remain relatively scarce. Available evidence indicates that both surgery and adjuvant chemotherapy elicit measurable inflammatory responses in BC patients, though with distinct temporal profiles. Surgical resection triggers an acute systemic response, most consistently marked by sharp increases in IL-6, but also TNF-α and IL-8, peaking within 6–24 h and typically resolving within days to weeks, except in cases of complications or concurrent treatments [6,17,18,19,20,21]. By contrast, chemotherapy induces more gradual and prolonged cytokine alterations. In postmenopausal women with early breast cancer, longitudinal studies reported short-term increases of 15–48% in IL-6, IL-8, IFN-γ, TNF-α, and IL-10 after adjuvant chemotherapy, returning toward baseline within 6–12 months [22]. Another important longitudinal study showed that patients receiving chemotherapy exhibited statistically significant increases in circulating TNF-α, sTNF-RII, IL-6, and IFN-γ from pre- to post-treatment. Those treated with combined chemotherapy and radiation additionally showed significant increases in IL-8, and elevations in TNF-α, sTNF-RII, IL-6, IFN-γ, and IL-8 persisted at 6, 12, and 18 months post-treatment [23]. Larger studies further associate chemotherapy-related increases in IL-6, TNF-α, and soluble TNF receptors with impaired physical function and fatigue (e.g., [24]), while smaller cohorts link IL-6 and MCP-1 elevations with cognitive decline (e.g., [25,26,27]). Across studies, IL-6 emerges as the most consistent biomarker, and for TNF-α, IL-8, IFN-γ, IL-10, evidence is more fragmented and heterogeneous, with quality ranging from low to moderate depending on the context. However, data should be interpreted with caution given the number of available studies and the heterogeneity in sample size, disease stage, treatment regimens, confounding variables related to symptoms or to concurrent therapeutic regimens during chemotherapy (e.g., corticosteroids and antiemetics), timing, menopausal status, metabolic status, and assays which limit extrapolation to clinical endpoint. In summary, there is consistent evidence, but it remains relatively limited in rigor and scope [4,10,15,22,23,24].

Third is stress response system activation. BC diagnosis and treatment constitute profound sources of chronic stress, precipitating cascades of stress-related psychological and physiological responses. Sustained activation of neuroendocrine and immune pathways disrupts homeostasis, elevates allostatic load, and fosters inflammation-driven tumorigenesis [28,29,30,31,32,33,34,35,36,37,38]. Persistent stress responses impair immunocompetence, exacerbate vulnerability to disease progression, and worsen psychological outcomes [23,29,34,36,37,38,39,40,41,42]. Indeed, chronic stress may be cancer-permissive, influencing disease onset and progression through both immunosuppressive and pro-inflammatory mechanisms [29,38,40,41,43,44,45,46,47,48,49]. In line with these mechanisms, positive and resilience-related factors may also exert neuroendocrine and immune effects [50]. Evidence indicates that intrinsic positive psychosocial processes—encompassing perceptual, cognitive, emotional, behavioral, and relational domains—may modulate a distinct immunophenotype, thereby buffering the deleterious effects of stress and influencing autonomic system responses [28,29,42,48,51,52,53,54,55,56,57,58,59,60,61,62,63]. Within this framework, coping strategies emerge as pivotal mediators. Adaptive, approach-oriented strategies (e.g., emotional expression and processing, positive reframing, acceptance, meaning-making, and spirituality) can mitigate stress, restore regulatory balance through neuroendocrine–immune regulation, and improve survival and well-being among BC patients, whereas maladaptive mechanisms (e.g., expressive suppression, rationalization, denial, and avoidance) intensify sympathetic activation, inflammatory cytokine release, and adverse health outcomes [48,52,64,65,66,67,68,69,70]. Collectively, these findings underscore the dual role of stress and coping in shaping cancer outcomes, highlighting coping as a determinant of resilience versus vulnerability across the breast cancer trajectory [29,42,46,48,52,70].

To contribute to a deeper understanding of the complexity of this equation, this study aims to investigate the immunological changes arising from the inflammatory footprint of anti-BC treatments and cancer-related psychosocial response. Specifically, it examines the impact of anticancer treatments on the inflammatory status of women with BC, analyses inflammatory and psychosocial processes throughout treatment trajectory, and evaluates the associations between inflammatory and psychosocial parameters following chemotherapy.

The analysis of our results highlights that BC treatments and psychosocial factors leave an identifiable inflammatory footprint in patients with BC; surgery and chemotherapy trigger different inflammatory profiles; coping influences inflammation via its function rather than through the specific strategy employed; stress and maladjusted coping trigger distinct deleterious inflammatory responses; and a biopsychosocial approach to cancer treatment and patient rehabilitation may enhance overall health outcomes.

## 2. Materials and Methods

### 2.1. Study Design

This longitudinal prospective cohort study was conducted in a breast cancer unit within a Portuguese cancer center, with the objectives of investigating the changes in cytokine plasma levels and examining the interplay between medical, psychosocial, and biological variables across two time points: (T1) post-diagnosis and before chemotherapy and (T2) shortly after the completion of adjuvant chemotherapy (see detailed study protocol [71]).

### 2.2. Study Population

A convenience sample of 106 women diagnosed with premenopausal BC at stages I, II, or III, scheduled to initiate chemotherapy at the ODC unit, were voluntarily recruited and assessed. The total sample was stratified into two sub-cohorts: one receiving adjuvant (post-surgery) treatment (*n* = 73), and the other undergoing neoadjuvant (pre-surgery) treatment (*n* = 33). Patient selection was conducted during the weekly multidisciplinary team meeting based on predefined criteria. All eligible participants were contacted by telephone and invited to participate.

Inclusion criteria: ≥18 years; premenopausal status; BC stage I to III at diagnosis; chemotherapy as part of the treatment protocol; able to speak, read, and write Portuguese fluently; and agreement and availability to participate after providing written informed consent according to local Ethical Committee (Ref. CES. CI-IPOP81/2017) and National Commission of Data Protection (CNPD; 13413/2017) approvals.

Exclusion criteria: previous history of cancer; past psychiatric history, including addictive disorders; past neurological history; presence of other severe medical comorbidities; and data storage (psychosocial or biological) refusal.

Detailed descriptions of the sample characteristics are presented in Table 1.

### 2.3. Materials and Measures

#### 2.3.1. Sociodemographic and Medical Data Form

Sociodemographic data included age, marital/relationship status, educational level, employment status, and occupational condition.

Disease and treatment-related information was obtained from medical record abstraction and included cancer site and stage, date of diagnosis, time since diagnosis, type of surgery received, type of adjuvant treatment received, and treatment protocol.

#### 2.3.2. Psychosocial Measures

Perceived Stress Scale (PSS; [72]; Portuguese 14-item version [73]). This standardized self-report questionnaire assesses the extent to which an individual perceives their life as stressful or experiences overall stress in response to stressful life events. Each item is rated on a 5-point Likert scale ranging from 0 (never) to 4 (very frequently), with a total score between 0 and 56. The original version [72] demonstrated a 0.86 α coefficient. In the present study, the α coefficient was 0.70 at T1 and 0.61 at T2. Higher scores indicate greater perceived levels of overall stress in daily life.

Brief Cope Inventory (Brief Cope; [74]; Portuguese version [75]). This inventory evaluates coping styles and strategies. The 28-item version was used, comprising 14 subscales, each with two items representing a coping style: active coping; planning; instrumental support; emotional support; religion; positive reframing; self-blaming; acceptance; venting; denial; self-distraction; behavioral disengagement; substance use; and humor [74,75]. The α coefficient ranged from 0.55 to 0.81 across the 14 subscales in the Portuguese version [75]. In the present study, a range from 0.46 to 0.93 was observed at T1, and a range from 0.20 to 0.93 was observed at T2. Higher subscale scores indicate greater utilization of the corresponding coping strategy.

Functional Assessment of Chronic Illness Therapy (FACIT; [76]). This questionnaire assesses perceived health-related quality of life in cancer patients. It comprises a general module (GWB) supplemented by cancer type-specific or cancer-related impact questionnaires [76]. This study included the Functional Assessment of Cancer Therapy—General Module (FACT-G; [75]), a multidimensional instrument measuring perceived quality of life (GWB) with 27 items distributed across four subscales: Physical Well-Being (PWB; 7 items), Social Well-Being (SWB; 7 items), Emotional Well-Being (EWB; 7 items), and Functional Well-Being (FWB; 6 items). In the current study, α coefficient values of 0.90 at T1 and 0.58 at T2 were observed. It also included the Functional Assessment of Cancer Therapy—Breast Cancer (FACT-B; [77]), a unidimensional measure consisting of 10 items specifically designed to assess BC-related concerns and impact. The FACT-B demonstrated α coefficients of 0.70 at T1 and 0.69 at T2. Both the FACT-G and FAT-B questionnaires utilize a 5-point Likert scale ranging from 0 (not at all) to 4 (very much), with higher scores indicating greater perceived quality of life [76,77].

#### 2.3.3. Biological Markers

Blood samples were collected to assess interleukins (IL)-2, IL-6, IL-17a, IL-4, and IL-10; tumor necrosis factor alpha (TNF-α); and interferon gamma (IFNγ).

### 2.4. Procedures

The research team consecutively selected 106 participants during the weekly BC multidisciplinary meetings. Eligible patients were then contacted by telephone to receive information about the study protocol and to assess their willingness to participate.

Subsequently, an appointment was scheduled during the routine pre-chemotherapy hospital visit, where the research protocol and procedures were explained in detail. Written informed consent was obtained from all participants. Following consent, the first blood sample was collected concurrently with the pre-treatment routine blood tests.

On the first day of chemotherapy, approximately 2 weeks after the initial appointment, participants completed the psychosocial measures.

One week before the second assessment (nearly two weeks after chemotherapy ended), participants were contacted and given instructions for the following data collection.

On the T2 evaluation day, blood samples were collected according to the same standardized procedures as in T1, followed by psychosocial measures.

Biological data from T1 and T2 were generated using classic/standardized, manual, and individualized laboratory methods. Approximately 3 mL of venous blood was drawn from each participant under strict aseptic conditions and properly labeled. Serum was separated by centrifugation at 1400 rpm for five minutes and then transferred into labeled cryotubes and stored at −70 °C until further analysis. Subsequently, the concentrations of pro-inflammatory cytokines (IL-2, IL-6, IL-17a, TNF-α, and IFNγ) and anti-inflammatory cytokines (IL-4 and IL-10) were measured from the stored serum samples using the BD™ Cytometric Bead Array (CBA) method on a FACSCanto™ II flow cytometer (BD Biosciences, Franklin Lakes, NJ, USA) following the manufacturer’s protocol. The flow cytometry data were analyzed using FCAP Array software, version 3.0 (BD Biosciences).

Additional methods and procedures are detailed in the published study protocol [71].

### 2.5. Statistical Analysis

Statistical analysis was managed with the SPSS^®^ v.27.

Data distribution was assessed using Descriptive Statistics, examining skewness (Sk), and kurtosis (K), following Kline’s criteria [78], with acceptable values for skewness (Sk) and kurtosis (K) being below 3.0 and 8.0, respectively. At T1, the scores of the instrumental support, behavioral disengagement, and substance use subscales of the Brief Cope Questionnaire and the plasma levels of cytokines IL-10, IL-6, and IL-4 did not meet the Sk and K criteria. At T2, the scores of the substance use and social/family well-being subscales and the plasma levels of cytokines IL-2, IL-6, and IFNγ similarly did not meet normality criteria. Despite these deviations, these data were retained for parametric analysis due to the expected intraindividual variability in physiological and psychosocial indicators inherent to complex medical conditions such as cancer. This variability reflects biological and psychosocial fluctuations related to disease course, treatment, and individual adjustment processes. Moreover, although the relatively small sample size may limit the assumption of normality, the findings align with prior research, indicating that distinct cancer treatments and psychosocial responses can elicit different inflammatory profiles. Therefore, the observed variability is biologically plausible [79,80,81].

Sociodemographic and clinical data, along with psychosocial and biological measures, were estimated using Descriptive Statistics.

Paired sample *t*-tests were conducted to compare psychosocial and immunological parameters across the total sample between the two timepoints. Subsequently, independent samples *t*-tests were performed to compare psychosocial and inflammatory variables within the adjuvant and neoadjuvant sub-cohorts at Time 1 and Time 2 evaluations.

The associations among the biomarkers and between the biomarkers and psychosocial indicators were analyzed using Pearson product–moment correlation tests.

## 3. Results

### 3.1. Descriptives from Time 1 and Time 2

The means and standard deviations for both psychosocial and inflammatory indicators at Time 1 (T1; before chemotherapy) and Time 2 (T2; after chemotherapy) are presented in Table 2.

### 3.2. Comparative Analyses Between Time 1 and Time 2 Assessments

#### 3.2.1. Comparative Analysis Between the Total Samples

The comparative analysis (c.f. Table 2) of the psychosocial variables showed a significant difference in instrumental support (*p* = 0.019), emotional support (*p* = 0.031), and religion (*p* = 0.015) Brief Cope subscales, with the first two scoring higher at T1 and the last one at T2; GWB (*p* = 0.011) and its PWB (*p* = 0.000) and FWB (*p* = 0.010) subscales showed significantly higher scores pre-chemotherapy; and the FACT-B (*p* = 0.000) score presented a significant decline in BC-specific quality of life issues post-chemotherapy.

Plasma levels of IL-17a (*p* = 0.000), TNF-α (*p* = 0.000), and IL-10 (*p* = 0.020) between T1 and T2 revealed a significant decrease. The analysis of the biological parameters demonstrated small effect sizes for IL-17a (0.40), TNF-α (0.37), and IL-10 (0.23), suggesting that the magnitude of these differences was relatively modest.

#### 3.2.2. Comparative Analysis Between the Adjuvant Subsamples

Paired sample *t*-tests comparing the study variables in the adjuvant group between T1 and T2 revealed statistically significant differences in perceived stress (*p* = 0.052), with higher scores observed post-diagnosis; the emotional support (*p* = 0.035) subscale, with higher scores following diagnosis; the GWB (*p* = 0.002) and its PWB (*p* < 0.001) subscale, with both quality of life indices presenting higher scores pre-treatments; and FACT-B (*p* < 0.001), which showed lower levels of BC-related quality of life post-chemotherapy.

The comparative analysis of the biomarkers identified significant differences in IL-17a (*p* < 0.001) and TNF-α (*p* = 0.026), both showing lower levels post-chemotherapy. IL-10, in contrast to the findings in the total sample, did not exhibit a significant difference.

#### 3.2.3. Comparative Analysis Between the Neoadjuvant Subsamples

The comparative analysis of the psychological variables between T1 and T2 in the neoadjuvant cohort revealed the following significant results: the instrumental support (*p* = 0.004) Brief Cope subscale had higher scores post-diagnosis; the religion (*p* = 0.027) and denial (*p* = 0.051) Brief Cope subscales had higher scores at T2; the PWB (*p* < 0.001) and FWB (*p* = 0.013) subscales were significant, with both quality of life parameters declining post-chemotherapy; and, FACT-B (*p* < 0.001), which showed decreased scores, indicating reduced BC-related quality of life at T2.

The analysis of differences in inflammatory markers between the two assessments revealed that circulating levels of IL-17a (*p* = 0.024), TNF-α (*p* = 0.003), and IL-10 (*p* = 0.010) were reduced in the periphery following chemotherapy.

### 3.3. Comparative Analyses Between Adjuvant and Neoadjuvant Cohorts

#### 3.3.1. Comparative Analysis at Time 1

The comparative analysis between patients undergoing adjuvant and neoadjuvant treatments at T1 found no significant differences between the groups, except for the self-blaming (*p* = 0.030), and humor (*p* = 0.042) subscales of the Brief Cope Questionnaire, which were higher in the adjuvant treatment cohort. The remaining variables showed no statistically significant differences, as indicated by a non-significant *p*-value and small effect sizes.

The analysis of the biomarkers revealed a significant difference in the circulating levels of TNF-α (*p* = 0.001) at T1, with a medium effect size, indicating that the neoadjuvant cohort had higher levels of this pro-inflammatory cytokine. The remaining biomarkers showed small effect sizes, further supporting the absence of differences between groups.

Detailed information regarding comparative analysis is provided in Appendix A.

#### 3.3.2. Comparative Analyses at Time 2

The comparative analysis between the adjuvant and neoadjuvant cohorts at T2 revealed that the instrumental support Brief Cope subscale was the only variable among the psychosocial and biological markers that showed a statistically significant difference (t = −2.357; 83.37; *p* = 0.021; d = 0.44), with the higher score observed in the adjuvant group [5.25 (1.58); 4.61 (1.14)].

The remaining psychosocial and biological parameters exhibit small effect sizes, confirming the similarities between the sub-cohorts post-chemotherapy.

Complete results from the independent sample *t*-tests are presented in Appendix B.

### 3.4. Bivariate Analysis

#### 3.4.1. Bivariate Analysis Between Inflammatory Biomarkers

The associations between the inflammatory markers post-chemotherapy in the total sample (N = 106) are displayed in Table 3. Interleukin-6 is the only cytokine that does not show a relationship with the other biomarkers. The remaining correlations between the inflammatory markers are all positive and statistically significant, with *p* < 0.001.

#### 3.4.2. Bivariate Analysis of Inflammatory and Psychosocial Markers at Time 2 in the Total Sample and Treatment Sub-Cohorts

Based on the biomarkers that showed significant differences pre- and post-chemotherapy, correlations were calculated between IL-17a, TNF-α, and IL-10 and the psychosocial indices, as presented in Table 4.

## 4. Discussion

**Figure 1:**
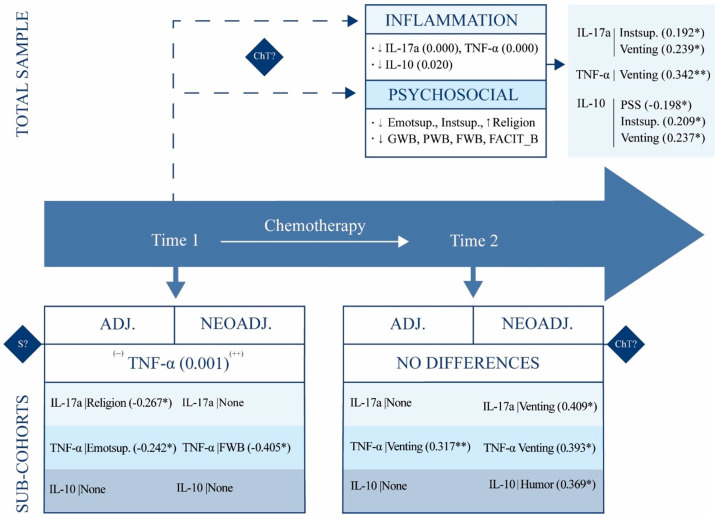
Interaction of psychosocial and biological factors within/between assessments. Note. PSS = Perceived Stress Scale; Instsup. = Instrumental Support; Emotsup. = Emotional Support; GWB = Functional Assessment of Cancer Therapy—General Well-Being Module; PWB = Physical Well-Being Subscale; FWB = Functional Well-Being Subscale; FACT_B = Functional Assessment of Cancer Therapy—Breast Cancer Module; ADJ. = Adjuvant cohort; NEOADJ. = Neoadjuvant cohort; IL = Interleukin; TNF-α = Tumor Necrosis Factor alpha; ↑ = increase; ↓ = decrease; (--) = lower; (++) = higher; * *p* < 0.05; ** *p* < 0.01.

The analysis of the total sample, along with the adjuvant and neoadjuvant cohorts, yield nuanced insights into the dynamics of inflammatory biomarkers and psychosocial factors before and after chemotherapy, as well as their interrelationship. The principal findings are summarized in Figure 1.

### 4.1. The Influence of Anti-BC Treatment on Inflammation and Immunomodulation

#### 4.1.1. Time 1

The significantly lower circulating levels of TNF-α observed in the adjuvant cohort compared with the neoadjuvant cohort at T1 may be attributed to prior surgical tumor resection. Surgical removal likely reduced TNF-α expression, resulting in decreased systemic levels relative to patients in the neoadjuvant group, who had not yet undergone surgery. According to Esquivel-Velázquez and colleagues [82], inflammation within the TME induces the secretion of cytokines, chemokines, and growth factors, which in turn modulate the inflammatory milieu and, when upregulated, promote cancer cell proliferation and invasion, thereby accelerating tumor progression [5]. TNF-α, a pro-inflammatory cytokine, is highly expressed in breast carcinomas, where its chronic expression fosters carcinogenesis and tumor growth [83]. Conversely, TNF-*α* inhibition exerts a protective effect against breast tumorigenesis [4,84], particularly during the early stages of carcinogenesis, including angiogenesis and invasion [4]. In vitro studies have further demonstrated that activation of the TNF-α axis induces invasive behavior in BC cells [85], while TNF-α more broadly promotes tumor progression by enhancing cellular migration within the TME [81,85]. Consistent with these findings, the present study suggests that, during the pre-chemotherapy assessment, TNF-α levels were highly responsive to surgical tumor removal, contributing to the lower inflammatory profile observed in the adjuvant group compared with the neoadjuvant group. Supporting this, Bulska-Będkowska and colleagues [18] reported that baseline serum concentrations of the soluble IL-6 receptor (sIL-6R) and the TNF-α receptor (sTNF-R1) were significantly higher prior to surgery than at the follow-up assessment two months later (*p* < 0.05), reinforcing the predictive value of surgery in breast cancer. Additionally, previous studies indicate that surgical resection typically elicits an acute systemic response peaking within 6–24 h [17,20,21]. Since our T1 assessment was performed pre-chemotherapy, several weeks after surgery in the adjuvant group, we hypothesize that the observed decrease in TNF levels reflects a long-term benefit of surgery rather than a transient acute response.

#### 4.1.2. From Time 1 to Time 2

The analysis of biomarker levels between T1 and T2 revealed that TNF-α, IL-17a, and IL-10 showed a statistically significant decrease following chemotherapy. Contrary to previous landmark studies developed by Lindholm and colleagues [22] and Bower and colleagues [23], our findings do not support a gradual and sustained increase in cytokine levels following chemotherapy. However, our results are consistent with those of Jabeen and colleagues [7], who reported that FEC treatment led to a global reduction in cytokine levels—particularly VEGF-A, IL-12, IP-10, and IL-10—with a more pronounced decrease in the bevacizumab (Bev) arm. In this group, patients achieving a pathological complete response (pCR) exhibited lower levels of VEGF-A, IFN-γ, TNF-α, and IL-4, and circulating cytokine profiles correlated with higher infiltration of cytotoxic CD8+ T cells, underscoring the potential predictive value of these markers and the need for further investigation. Our findings suggest that chemotherapy alone, when administered after surgical intervention in the adjuvant group, promotes a beneficial reduction in TNF-α, IL-17a and IL-10 levels, likely reflecting extensive tumor cell death induced by anticancer therapies and the consequent attenuation of inflammatory mechanisms [86,87]. Moreover, IL-17, a key cytokine produced by T helper 17 (Th17) cells, is highly expressed in breast tumor tissue [12]. The upregulation of Th17 cells correlates with elevated IL-17 levels and has been associated with greater tumor aggressiveness through the induction of angiogenic factors in patients with BC [9]. The observed reduction in serum IL-17a levels in this cohort is consistent with the findings of Jabeen and colleagues [7], Karpisheh and colleagues [8], and Tzang and colleagues [9], that chemotherapy and anti-angiogenic therapies modulate immune responses during BC treatment, thereby underscoring the potential immunosuppressive effect of chemotherapy. Furthermore, these findings support the role of IL-17a as a prognostic marker [8]. According to Jabeen and colleagues [7], IL-17a is a hallmark of Th17 immunoactivity, functioning as a pro-inflammatory cytokine that stimulates the release of inflammatory and angiogenic growth factors, and it is also associated with resistance to anti-angiogenic therapies.

The decrease in IL-10 levels in T2 may be explained by the coordinated and synchronized roles of multiple cytokines, including IFN-α, INF-β, INF-γ, IL-2, IL-6, IL-8, TNF-α, and IL-10 in breast carcinogenesis [12,88,89], as reflected in the data presented in Table 3. IL-10 exhibits a complex, multifunctional role, exerting immunosuppressive and antiangiogenic effects while also displaying dual proliferative and inhibitory impacts on breast tumor cells [82,88,89,90]. Several studies have demonstrated that IL-10 inhibits inflammatory immune signaling by downregulating pro-inflammatory cytokine expression, thereby acting as an antitumoral cytokine in certain cancers [4,12]. However, elevated IL-10 levels in the serum of BC patients may facilitate tumor immune evasion, as well as tumor growth and proliferation within the TME [89,90,91]. Moreover, high serum concentrations of IL-10, IL-6 and TNF-α have been associated with poorer survival outcome and increased resistance to the cytotoxic effects of chemotherapy [82,88]. In line with these findings, the observed decline in IL-10 levels post-chemotherapy may, similar to the reduction in other cytokines, represent a favorable regulatory shift in the inflammatory response of BC patients.

#### 4.1.3. Time 2

Given the absence of statistically significant differences between the adjuvant and neoadjuvant groups at T2, it may be hypothesized that the convergence of their inflammatory profiles is largely attributable to chemotherapy administered over the preceding months. At baseline, all inflammatory markers were statistically comparable between the two sub-cohorts, except for TNF-α, suggesting that surgery exerted a distinctive effect on TNF-α at T1. By T2, however, the inflammatory profiles of both groups were similar, most reflecting the impact of anticancer therapies on the TME and the extensive tumor cell death they induce [82,87].

In conclusion, the data indicates that TNF-α was the most sensitive biomarker to surgical resection in the adjuvant group prior to the T1 evaluation, underscoring the importance of local treatment. At T2, TNF-α and IL-17a emerged as the most sensitive biomarkers to chemotherapy across the total sample as well as within both sub-cohorts, reinforcing the role of systemic treatment. Additionally, IL-10 appeared more sensitive to chemotherapy in neoadjuvant patients, likely owing to its stronger correlation with TNF-α (c.f. Table 3), which was significantly higher in the neoadjuvant group at T1.

### 4.2. Longitudinal Analysis of Psychosocial Variables from Time 1 to Time 2

According to Lacourt and colleagues [92], stress levels are typically elevated following a cancer diagnosis and before treatment initiation, decline sharply during the first three months of treatment, and rise again between months 6 and 10. This pattern helps explain the absence of significant differences in perceived stress before and after chemotherapy in total sample, as well as the significant reduction in stress observed in the adjuvant group (but not in the neoadjuvant group), coinciding with the completion of the most aggressive treatment phase. This reduction may also be linked to the decreased reliance on instrumental and emotional support coping strategies after chemotherapy. Cancer, as a life-threatening event, initially mobilizes the patient’s support network during the early treatment stage [93]. Over time, however, stress levels tend to decrease [92], potentially due to several factors: (a) the transition from a crisis period to one of readjustment; (b) a growing sense of control and mastery; and (c) the realization that, despite challenges, the individual is coping more effectively than expected. As patients regain functionality in daily life, significant others may alter their attitudes, reducing the level of support they initially provided [94]. When stress levels rise again [92], often due to the persistence of chronic stressors, the social support network may have already diminished or shifted focus toward its own concerns rather than the patient’s needs, a process described by the “erosion model” [95]. The reduced reliance on external sources of support may foster greater use of internal coping strategies, such as religious coping. Toledo and colleagues [96] reported that this coping strategy can help women with BC adjust psychosocially to the disease, improving treatment adherence, managing side-effects, and providing a sense of purpose and meaning. Finally, perceived well-being may significantly decline as a consequence of the psychobiological side effects of both the tumor and its treatment, resulting in reduced overall quality of life, particularly in physical, functional, and BC-specific domains [97,98,99].

### 4.3. The Interplay Between Psychosocial and Biological Factors in BC

#### 4.3.1. Perceived Stress and Inflammation

The literature highlights the deleterious effects of sustained high stress levels on the inflammatory response in cancer patients [28,29,40,41,50,70]. Our study corroborates these findings, revealing a negative correlation between perceived stress and the anti-inflammatory cytokine IL-10 in the total sample post-chemotherapy. These results indicated that elevated stress compromises the anti-inflammatory response, emphasizing the adverse impact of stress on immune function.

#### 4.3.2. Coping and Inflammation

The coping strategies of venting and emotional and instrumental support emerged as the most significant variables in the correlation analyses with biomarkers and will therefore be highlighted.

##### Venting

Our data showed significant positive correlations between venting and inflammation across the total sample, as well as within the neoadjuvant and adjuvant sub-cohorts (c.f. Table 4 and Figure 1). These associations may be consistent with the notion that the effectiveness of a coping strategy depends primarily on its function and the intention underlying its use, rather than on the strategy itself. Our findings suggest that emotional expression via venting is likely not serving an adjustment-promoting function. This hypothesis is supported by the positive correlations observed between venting and (a) higher levels of perceived stress [r = 0.268, *p* < 0.01]; (b) experiential avoidance coping strategies, including denial [r = 0.231, *p* < 0.05] and planning [r = 0.324, *p* < 0.01]); and (c) greater reliance on external rather than internal resources, specifically emotional [r = 0.323, *p* < 0.01] and instrumental [r = 0.249, *p* < 0.01] support. Additionally, venting was negatively associated with emotional well-being (EWB [r = −0.280, *p* < 0.01]). These results suggest that venting is not effectively promoting internally focused emotional processing or facilitating adaptive management of the cancer experience. Supporting this interpretation, Hoyt and colleagues [100], in a study of prostate cancer patients, found that emotional processing predicted lower levels of inflammation, whereas emotional expression, when not coupled with emotional processing, was associated with increased inflammation. The authors concluded that expressing emotions without attempting to derive meaning from the experience may contribute to dysregulation and promote maladaptive cognitive patterns (e.g., rumination, persistent worry, and repetitive negative thinking), which in turn influence inflammatory processes.

##### Instrumental and Emotional Support

Although social support is generally associated with protective health outcomes, the observed decline in the use of instrumental and emotional support across the cancer trajectory may reflect patients’ willingness to seek support rather than the absence of available support (e.g., items “I ask for…”; “I seek for…”). In the total sample, the significant reduction in emotional and instrumental support use indicates that “having support” (perceived support) and “utilizing support” (coping strategy) involve distinct psychological processes, highlighting the difference between choosing to seek support and feeling the need to do so.

The cancer-related need for support, combined with the mechanisms described in the “erosion model” [95], likely accounts for the positive correlations observed between the support-based coping strategies and IL-17a and IL-10 at T2 in the total sample. Conversely, the negative association between emotional support and TNF-α at T1 in the adjuvant cohort [r = −0.242, *n* = 73, *p* < 0.05] suggests a protective role of this strategy against inflammation. This effect may be attributed to the critical role of emotional support during the initial crisis period following diagnosis and surgery, when it mitigates stress and enhances perceived control and self-efficacy, in contrast to later stages of the cancer trajectory.

## 5. Conclusions

Our findings suggest an inflammatory footprint shaped by both anti-BC treatments and psychosocial factors in women undergoing chemotherapy.

In our sample, TNF-α was the only cytokine highly sensitive to surgical intervention, highlighting the role of local treatments in reducing TME inflammation. Furthermore, TNF-α, IL-17a, and IL-10 levels significantly decreased post-chemotherapy, indicating that these cytokines are particularly responsive to the systemic effects of chemotherapy. Moreover, after chemotherapy, both adjuvant and neoadjuvant cohorts displayed similar inflammatory profiles, further supporting the immunoregulatory effect of chemotherapy. Our findings regarding breast cancer surgery are consistent with the existing literature, suggesting that, following an immediate increase in circulating cytokine levels, these levels subsequently tend to decrease. Concerning the impact of chemotherapy on circulating cytokine levels, although our results are consistent with those reported by Jabeen and colleagues [7], they do not corroborate the main body of existing literature, as we did not observe an increase in the pro-inflammatory response following chemotherapy. These discrepancies highlight the need for caution in interpretation, given the heterogeneity of breast cancer and population characteristics (e.g., disease characteristics and stages, race, age, and menopausal status, with most landmark studies having been conducted in postmenopausal or mixed groups). Additional variability arises from differences in treatment protocols, concomitant therapies, and symptom profiles. Current evidence is further limited by the scarcity of large prospective cohorts with standardized cytokine panels and predefined collection time points, the lack of analytic standardization, and the absence of RCTs or quasi-experimental designs assessing the interplay between cancer treatments, cytokine levels, and clinical endpoints. To advance the field, pre-registered meta-analyses with standardized temporal windows, log-transformed values, and robust methods for censored data, as well as rigorous control of key covariates (e.g., corticosteroid use, infections, BMI, menopausal status, and chemotherapy regimen), are warranted.

Our results also indicate that the psychosocial component of the observed inflammatory footprint is particularly shaped by coping strategies, namely venting, emotional and/or instrumental support. These strategies were positively associated with the three inflammatory biomarkers that decreased post-chemotherapy in the total, neoadjuvant, and adjuvant samples. TNF-α demonstrated sensitivity to the venting coping strategy in the total and subsamples, with the impact of psychosocial factors being more pronounced in the total sample and the neoadjuvant cohort. However, it is important to consider that neoadjuvant treatment is typically administered for larger tumor masses, which may also influence overall TNF-α concentrations. Additionally, with respect to coping strategies, our findings highlight the importance—both clinically and from a research perspective—of analyzing the effects of coping strategies on overall well-being and health outcomes in light of the adaptive functions they fulfill, rather than focusing solely on the specific coping strategies themselves.

The responsiveness of inflammatory mechanisms to anti-BC treatments, along with the influence of psychological adjustment factors, highlight the importance of a biopsychosocial approach to cancer care. Future research should further elucidate the complex interaction between the protective and risk factors of both physiological and psychological processes involved in cancer-related inflammation.

## Figures and Tables

**Table 1:** Sociodemographic and health status characteristics (N = 106).

	M	SD	Min.	Max.	N	(%)
Sociodemographic variables						
Age (years)	42.09	5.45	27	51		
Marital Status						
Single					9	8.5
Married					71	67.0
Divorced					9	8.5
Partnership					17	16.0
Education Level (years)	11.28	4.54	1	22		
Profession						
Qualified job					32	30.2
Non-qualified job					64	60.4
Unemployed					3	2.8
Housewife					7	6.6
Professional Status						
At work					21	19.8
On sick leave					75	69.6
Unemployed/housewife					10	10.6
Medical information						
Time since diagnosis (months)	3.27	1.58	1	8		
Disease Stage						
I					2	1.9
II					43	40.6
III					61	57.5
Type of Treatment						
Neoadjuvant					33	31.2
Adjuvant					73	68.8

**Table 2:** Means and standard deviations of psychosocial and inflammatory markers at T1 and T2 and results of paired sample *t*-test (N = 106).

	T1: BChT		T2: AChT		T1-T2 Comparison			
	*M*	*SD*	*M*	*SD*	*t*	*df*	*p*	*d*
Psychosocial variables								
PSS	35.97	6.07	35.04	5.51	1.628	104	0.114	0.159
BCOPE ActiveC. Plan. Emotsup. Instsup. Religion Positref. Sblaming Accepta. Venting Denial Sdistrac. Bhdiseng. Subuse. Humor	6.30 5.50 6.20 5.63 4.79 5.49 2.58 6.77 4.07 2.71 6.18 2.20 2.09 4.42	1.33 1.50 1.61 2.38 2.14 1.75 1.02 1.18 1.46 1.06 1.34 0.54 0.97 2.07	6.25 5.41 5.79 5.05 5.21 5.60 2.53 6.78 3.93 2.84 6.02 2.17 2.03 4.60	1.25 1.43 1.67 1.48 2.09 1.57 0.89 1.19 1.30 1.17 1.63 0.49 0.22 2.05	0.321 0.564 2.191 2.374 −2.481 −0.609 0.541 −0.071 0.815 −0.886 0.859 0.491 0.681 −1.026	104 105 105 105 105 105 105 105 105 105 105 105 105 105	0.749 0.574 0.031 0.019 0.015 0.544 0.589 0.943 0.417 0.378 0.392 0.624 0.497 0.310	0.031 0.055 0.213 0.231 −0.241 −0.059 0.053 −0.007 0.079 −0.086 0.083 0.048 0.066 −0.099
FACIT-G GWB PWB SWB EWB FWB	85.83 26.08 21.35 18.65 19.75	12.16 2.72 4.29 4.32 4.43	82.18 22.02 22.38 19.26 18.52	15.83 5.11 10.16 3.65 4.71	2.575 8.396 −1.023 −1.691 2.623	105 105 105 105 105	0.011 0.000 0.309 0.094 0.010	0.250 0.815 −0.099 −0.164 0.255
FACT-B	31.20	5.36	27.21	9.92	6.500	105	0.000	0.631
Inflammatory biomarkers								
IL-17a IFNγ TNF-α IL-10 IL-6 IL-4 IL-2	14.73 1.02 1.86 2.16 5.80 3.50 0.89	12.17 1.50 1.90 3.42 28.51 15.55 1.18	8.67 0.74 0.99 1.34 5.66 0.95 1.02	10.13 1.34 1.42 1.70 26.78 1.26 1.54	4.140 1.478 3.777 2.360 0.164 1.674 −0.845	104 105 105 104 105 105 102	0.000 0.142 0.000 0.020 0.870 0.097 0.400	0.402 0.144 0.367 0.230 0.016 0.163 −0.083

Note. d = Cohen’s d; BChT = Before Chemotherapy; AChT = After Chemotherapy; PSS = Perceived Stress Scale; BCOPE = Brief Cope Questionnaire; ActiveC = Active Coping; Plan. = Planning; Instsup. = Instrumental Support; Emotsup. = Emotional Support; Positref. = Positive Reframing; Sblaming = Self-Blaming; Accepta. = Acceptance; Sdistrac. = Self-Distraction; Bhdiseng. = Behavioral Disengagement; Subuse. = Substance Abuse; FACIT = Functional Assessment of Chronic Illness Therapy; GWB = Functional Assessment of Cancer Therapy—General Well-Being Module; PWB = Physical Well-Being Subscale; SWB = Social/Family Well-Being Subscale; EWB = Emotional Well-Being Subscale; FWB = Functional Well-Being Subscale; FACT_B = Functional Assessment of Cancer Therapy—Breast Cancer Module; IL = Interleukins; IFNγ = Interferon gamma; TNF-α = Tumor Necrosis Factor alpha.

**Table 3:** Pearson product–moment correlations between inflammatory biomarkers at Time 2.

	IL-17a	IFNγ	TNF-α	IL-10	IL-6	IL-4	IL-2
IL-17a							
IFNγ	0.726 **						
TNF-α	0.673 **	0.861 **					
IL-10	0.600 **	0.728 **	0.706 **				
IL-6	0.022	0.074	0.116	0.028			
IL-4	0.668 **	0.682 **	0.650 **	0.528 **	−0.013		
IL-2	0.603 **	0.730 **	0.668 **	0.710 **	−0.012	0.582 **	

Note. IL = Interleukins; IFNγ = Interferon gamma; TNF-α = Tumor Necrosis Factor alpha. ** *p* < 0.01.

**Table 4:** Pearson product–moment correlations between inflammatory and psychosocial variables at Time 2.

	Total Sample (*N* = 106)	Adjuvant Cohort (*n* = 73)	Neoadjuvant Cohort (*n* = 33)
IL-17a	▪ Instrumental support (BCOPE) [*r* = 0.192, *p* < 0.05] ▪ Venting (BCOPE) [*r* = 0.239, *p* < 0.05]	▪ None	▪ Venting (BCOPE) [*r* = 0.409, *p* < 0.05]
TNF-α	▪ Venting (BCOPE) [*r* = 0.342, *p* < 0.01]	▪ Venting (BCOPE) [*r* = 0.317, *p* < 0.01]	▪ Venting (BCOPE) [*r* = 0.393, *p* < 0.05]
IL-10	▪ Perceived stress (PSS) [*r* = −0.198, *p* < 0.05] ▪ Instrumental support (BCOPE) [*r* = 0.209, *p* < 0.05] ▪ Venting (BCOPE) [*r* = 0.237, *p* < 0.05]	▪ None	▪ Humor (BCOPE) [*r* = 0.369, *p* < 0.05]

Note. PSS = Perceived Stress Scale; BCOPE = Brief Cope Questionnaire. IL = Interleukins; IFNγ = Interferon gamma; TNF-α = Tumor Necrosis Factor alpha.

## Data Availability

Access to data will be provided upon reasonable request.

## Abbreviations

The following abbreviations are used in this manuscript:

Accepta.	Acceptance coping
AchT	After chemotherapy
ActiveC.	Active coping
BC	Breast cancer
BChT	Before chemotherapy
BCOPE	Brief Cope Questionnaire
Bhdiseng.	Behavioral disengagement coping
CNS	Central nervous system
CRP	C-reactive Protein
Emotsup.	Emotional support coping
EWB	Emotional well-being
FACIT	Functional Assessment of Chronic Illness Therapy questionnaire
FACT-B	Functional Assessment of Cancer Therapy—Breast Cancer questionnaire
FACT-G	Functional Assessment of Cancer Therapy—General Module questionnaire
FWB	Functional well-being
GWB	General well-being
IFNγ	Interferon gamma9
IL	Interleukin
Instsup.	Instrumental support coping
K	Kurtosis
ODC	Oncology day care unit
Plan.	Planning coping
Positref.	Positive reframing coping
PSS	Perceived Stress Scale
PWB	Physical well-being
Sblaming	Self-blaming coping
Sdistrac.	Self-distraction coping
Sk	Skewness
Subuse.	Substance use coping
SWB	Social well-being
TGF-β	Transforming growth factor beta
Th	T helper cells
TME	Tumor microenvironment
TNF-α	Tumor necrosis factor alpha

## Appendix A

Means and standard deviations of study variables in adjuvant (*n* = 73) and neoadjuvant (*n* = 33) cohorts and independent sample *t*-test values at Time 1.

	***t*** **Student**							
	**Adjuvant** **Cohort** **(*n* = 73)**		**Neoadjuvant Cohort** **(*n* = 33)**					
	***M***	***SD***	***M***	***SD***	***t***	***df***	***p***	***d***
Psychosocial markers								
PSS Total Score	36.10	6.36	35.76	5.37	−0.266	104	0.791	−0.056
BCOPE ActiveC. Plan. Instsup. Emotsup. Religion Positref. Sblaming Accepta. Venting Denial Sdistrac. Humor	6.33 5.53 5.66 6.18 4.96 5.64 2.72 6.70 4.10 2.78 6.10 4.70	1.31 1.57 2.70 1.65 2.10 1.76 1.10 1.24 1.49 1.12 1.36 2.02	6.21 5.42 5.58 6.24 4.42 5.15 2.30 6.94 4.00 2.58 6.36 3.82	1.39 1.35 1.48 1.54 2.21 1.72 0.77 1.03 1.39 0.90 1.32 2.07	−0.432 −0.348 −0.163 0.189 −1.19 −1.34 −2.20 0.97 −0.31 −0.92 0.94 −2.06	103 104 104 104 104 104 85.76 104 104 104 104	0.667 0.729 0.871 0.850 0.236 0.182 0.030 0.334 0.755 0.358 0.345 0.042	0.091 −0.073 −0.034 0.040 −0.250 −0.282 −0.406 0.204 −0.066 −0.194 0.199 −0.433
FACT-G GWB PWB SWB EWB FWB	86.34 25.99 21.60 19.05 19.70	12.46 2.60 4.39 4.15 4.64	84.69 26.30 20.79 17.76 19.85	11.56 2.99 4.08 4.61 4.00	−0.643 0.554 −0.904 −1.44 0.161	104 104 104 104 104	0.521 0.581 0.368 0.153 0.873	−0.135 −0.116 −0.190 −0.302 0.034
FACT-B Total Score	30.89	5.57	31.88	4.85	0.879	104	0.382	0.184
Inflammatory biomarkers								
IL-17a	13.81	11.11	16.78	14.21	1.06	50.40	0.293	0.245
IFNγ	0.99	1.35	1.08	1.81	0.27	104	0.786	0.057
TNF-α	1.54	1.70	2.57	2.13	2.68	104	0.001	0.561
IL-10	2.11	1.78	2.29	2.52	0.25	104	0.806	0.052
IL-6	6.90	34.31	3.35	2.95	−0.59	104	0.555	−0.123
IL-4	4.29	18.68	1.75	1.91	−0.78	104	0.439	−0.163
IL-2	0.81	1.22	1.06	1.09	0.98	102	0.331	0.207

Note. d = Cohen’s d; PSS = Perceived Stress Scale; BCOPE = Brief Cope; ActiveC = Active Coping; Plan. = Planning; Instsup. = Instrumental Support; Emotsup. = Emotional Support; Positref. = Positive Reframing; Sblaming = Self-Blaming; Accepta. = Acceptance; Sdistrac. = Self-Distraction; FACIT = Functional Assessment of Chronic Illness Therapy; GWB = Functional Assessment of Cancer Therapy—General Well-Being Module; PWB = Physical Well-Being Subscale; SWB = Social/Family Well-Being Subscale; EWB = Emotional Well-Being Subscale; FWB = Functional Well-Being Subscale; FACT_B = Functional Assessment of Cancer Therapy—Breast Cancer Module; IL = Interleukins; IFNγ = Interferon gamma; TNF-α = Tumor Necrosis Factor alpha.

## Appendix B

Means and standard deviations of study variables in adjuvant (n = 73) and neoadjuvant (*n* = 33) cohorts and independent sample *t*-test values at Time 2.

	***t*** **Student**							
	**Adjuvant** **Cohort** **(*n* = 73)**		**Neoadjuvant Cohort** **(*n* = 33)**					
	***M***	***SD***	***M***	***SD***	***t***	***df***	***p***	***d***
Psychosocial markers								
PSS Total Score	34.61	5.20	35.82	6.15	1.041	103	0.300	0.219
BCOPE ActiveC. Plan. Instsup. Emotsup. Religion Positref. Sblaming Accepta. Venting Denial Sdistrac. Bhdiseng. Subuse. Humor	6.29 5.38 5.25 5.71 5.26 5.70 2.53 6.78 3.95 2.74 6.05 2.14 2.00 4.68	1.25 1.41 1.58 1.70 2.08 1.56 0.78 1.17 1.23 1.03 1.67 0.45 0.00 2.05	6.21 6.45 4.61 5.97 5.12 5.39 2.52 6.79 3.91 3.06 5.94 2.24 2.09 4.42	1.29 1.48 1.14 1.63 2.13 1.58 1.09 1.24 1.44 1.43 1.56 0.56 0.38 2.05	−0.285 0.236 −2.357 0.732 −0.316 −0.927 −0.102 0.028 −0.132 1.157 −0.335 1.032 1.359 −0.606	104 104 83.37 104 104 104 85.76 104 104 47.43 0.104 104 32.00 104	0.776 0.814 0.021 0.466 0.753 0.356 0.919 0.928 0.895 0.253 0.738 0.305 0.184 0.546	−0.060 0.050 −0.439 0.154 −0.066 −0.194 −0.021 0.006 −0.028 0.275 −0.070 0.216 0.426 −127
FACT-G GWB PWB SWB EWB FWB	82.03 21.93 21.58 19.55 18.97	13.50 5.49 4.13 3.42 4.66	82.52 22.21 24.15 18.64 17.72	20.29 4.23 17.19 4.11 4.52	0.146 0.260 1.212 −1.193 −1.1485	104 104 104 104 104	0.884 0.795 0.228 0.236 0.141	0.031 0.055 0.254 −0.250 −0.312
FACT-B Total Score	27.10	6.44	17.45	4.65	0.288	104	0.774	0.060
Inflammatory biomarkers								
IL-17a	8.63	9.89	8.75	10.81	0.054	104	0.957	0.011
IFNγ	0.69	1.26	0.83	1.53	480	104	0.632	0.101
TNF-α	1.01	1.49	0.94	1.48	−0.240	104	0.811	−0.050
IL-10	1.36	1.64	1.31	1.87	−0.142	103	0.888	−0.030
IL-6	6.95	31.98	2.83	6.30	−0.732	104	0.466	−0.153
IL-4	0.86	1.19	1.16	1.42	1.126	104	0.259	0.238
IL-2	1.20	1.81	0.81	1.45	−1.087	103	0.280	−0.230

Note. d = Cohen’s d; PSS = Perceived Stress Scale; BCOPE = Brief Cope; ActiveC = Active Coping; Plan. = Planning; Instsup. = Instrumental Support; Emotsup. = Emotional Support; Positref. = Positive Reframing; Sblaming = Self-Blaming; Accepta. = Acceptance; Sdistrac. = Self-Distraction; Bhdiseng. = Behavioral Disengagement; Subuse. = Substance use; FACIT = Functional Assessment of Chronic Illness Therapy; GWB = Functional Assessment of Cancer Therapy—General Well-Being Module; PWB = Physical Well-Being Subscale; SWB = Social/Family Well-Being Subscale; EWB = Emotional Well-Being Subscale; FWB = Functional Well-Being Subscale; FACT_B = Functional Assessment of Cancer Therapy—Breast Cancer Module; IL = Interleukins; IFNγ = Interferon gamma; TNF-α = Tumor Necrosis Factor alpha.
